# Inflammation and necrosis of the hepatic round ligament in a postpartum patient: a rare case report

**DOI:** 10.1093/jscr/rjag596

**Published:** 2026-07-16

**Authors:** Ioannis Arvanitis, Maria Galanopoulou, Nikolaos Siasos, Georgios Tzanoudakis

**Affiliations:** Department of Surgery, General Hospital of Agrinio, 3rd km Agrinio–Antirrio National Road, 30100 Agrinio, Greece; Department of Surgery, General Hospital of Agrinio, 3rd km Agrinio–Antirrio National Road, 30100 Agrinio, Greece; Department of Surgery, General Hospital of Agrinio, 3rd km Agrinio–Antirrio National Road, 30100 Agrinio, Greece; Department of Surgery, General Hospital of Agrinio, 3rd km Agrinio–Antirrio National Road, 30100 Agrinio, Greece

**Keywords:** hepatic round ligament necrosis, ligamentum teres hepatis, acute abdomen, cholelithiasis, postpartum complication

## Abstract

Hepatic round ligament necrosis is a rare etiology of acute abdomen and should be considered in unexplained right upper quadrant pain when common biliary pathology is absent. Preoperative diagnosis is challenging, and confirmation is usually intraoperative. We report the case of a 28-year-old postpartum female presenting twice to our emergency department with recurrent biliary colic. Initially, the patient was diagnosed with uncomplicated cholelithiasis and was discharged. Two weeks later, she returned with persistent biliary colic symptoms and biochemical abnormalities and was scheduled for laparoscopic cholecystectomy. Intraoperatively, extensive inflammation unrelated to the gallbladder necessitated conversion to open surgery. The patient underwent open cholecystectomy and round ligament resection. Histopathology confirmed necrosis with inflammatory infiltration of the ligament. Laboratory readings normalized postoperatively, and the patient was discharged on Day 3. We hypothesize that ascending infection related to postpartum omphalitis, along with pregnancy-related hypercoagulability, may have contributed to HRL necrosis.

## Introduction

The hepatic round ligament (HRL) is a fibrous remnant of the fetal umbilical vein. The veins associated with the ligament connect the portal venous system to the paraumbilical areas of the abdominal wall, where they drain into systemic veins. Only a limited number of cases of isolated HRL necrosis have been reported worldwide. According to existing research, the majority of patients present with right upper quadrant or epigastric pain, often mimicking biliary pathology, particularly biliary colic, acute cholecystitis, or pancreatitis [1–5]. Preoperative diagnosis remains elusive owing to the absence of pathognomonic imaging findings and low clinical awareness [4–6].

## Case presentation

A 28-year-old woman presented to our emergency department (ED) with sudden onset, continuous right upper quadrant abdominal pain. She was 2 months postpartum after an uncomplicated C-section. Her past medical history was unremarkable. She reported repeated instances of malodorous purulent umbilical discharge following her caesarean section, though she did not seek medical attention. On initial evaluation, her vitals and laboratory values were normal. An abdominal ultrasound revealed gallstones without wall thickening. The biliary colic was managed conservatively and she was discharged.

Two weeks after her initial presentation, the patient returned with localized tenderness on the right upper quadrant. Her full blood count showed leukocytosis (white blood cell count 13.5 × 10^9^/l) with neutrophilia. From the biochemical studies, she had a mildly elevated C-reactive protein of 1.9 mg/dl and abnormal liver function tests, including a total bilirubin: 2.8 mg/dl (direct 1.1 mg/dl), an aspartate aminotransferase of 157 U/l, alanine aminotransferase of 617 U/l, gamma-glutamyl transferase of 92 U/l, alkaline phosphatase of 176 U/l, and amylase of 176 U/l. The abdominal ultrasound revealed no evidence of acute cholecystitis or biliary obstruction. She was admitted for IV fluids, analgesics, and prophylactic antibiotics. A laparoscopic cholecystectomy was scheduled for the following day. Upon admission, a magnetic resonance cholangiopancreatography (MRCP) was carried out to examine the biliary tree, confirming cholelithiasis with normal cystic and common bile duct (CBD) diameters (Fig. 1).

**Figure 1:**
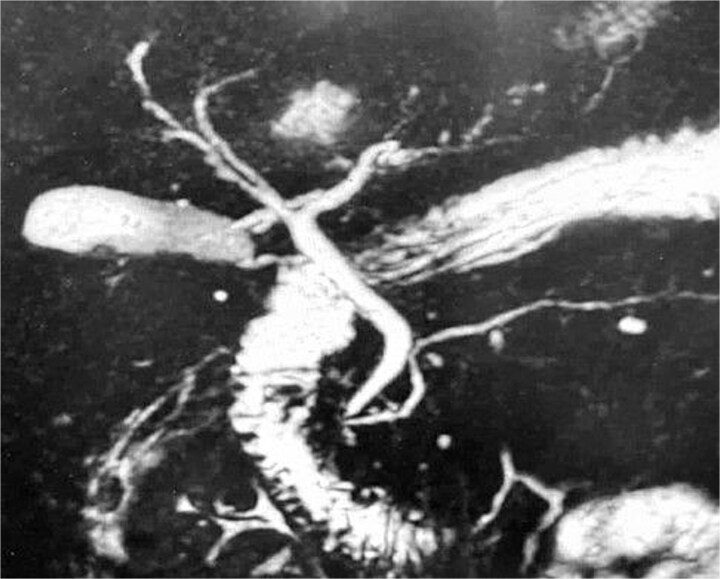
MRCP demonstrating normal cystic duct and common bile duct diameters without biliary tree dilatation or evidence of choledocholithiasis.

During surgery and upon placement of umbilical and subxiphoid trocars, there was extensive inflammation that distorted the normal anatomy, as well as several caesarean-related adhesions. Due to the risk of bile duct injury and the inability to recognize a specific pathology that could account for the diffuse inflammation in the presence of a macroscopically normal gallbladder, the procedure was converted from laparoscopic to open. The HRL was eventually identified, with a severely thickened, inflamed, and necrotic appearance. A cholecystectomy was performed, followed by complete excision of the necrotic ligament. The histopathology report demonstrated chronic cholecystitis changes, as well as extremely severe acute suppurative inflammatory infiltration with associated fat necrosis of enzymatic type from the HRL specimen. The postoperative course was uneventful and laboratory readings normalized within days following surgery. She was discharged on postoperative day 3 and remained asymptomatic at 3-month follow-up.

## Discussion

Necrosis of the HRL is a rare and underappreciated surgical condition. According to the existing literature [3–5], this pathology affects predominantly female patients, usually of middle age, and is characterized by acute abdominal pain in the right upper quadrant or epigastrium. The clinical picture is typically nonspecific, frequently mimicking biliary colic, acute cholecystitis, or acute pancreatitis, which is why most patients are initially evaluated for a common biliary pathology. Importantly, the abnormal biochemical profile observed in this patient with elevated bilirubin, transaminases, and amylase could not be directly explained by the HRL necrosis itself. Laboratory abnormalities are often non-diagnostic and insignificant unless there is a coexisting hepatobiliary disorder. In the setting of the patient’s gallbladder dysfunction, the preoperative workup is most likely attributable to transient biliary obstruction from microlithiasis, with HRL necrosis representing a concurrent but independent process. All of these laboratory abnormalities can resolve rapidly following cholecystectomy and may occur even when MRCP fails to identify ductal stones [7].

The relative contribution of symptomatic gallstone disease and HRL necrosis to the patient’s presentation remains difficult to ascertain. While the biochemical abnormalities were more consistent with transient biliary obstruction related to gallstone disease, the extensive inflammatory, and necrotic changes of the ligament identified intraoperatively and confirmed histologically indicate that HRL necrosis was not merely an incidental finding. Given the severity of the local inflammatory process and its impact on the operative strategy, we believe that the patient’s presentation was likely multifactorial, with both pathologies contributing to the overall clinical picture.

Preoperative recognition of HRL necrosis remains difficult. Because of the condition’s rarity and lack of clinical awareness, related imaging findings from abdominal ultrasound or computed tomography (CT), even if found, are often missed or misinterpreted. As a result, the definitive diagnosis is usually established intraoperatively during surgical exploration for suspected biliary disease. In our instance, the MRCP had already excluded obstruction, and the key clinical question of whether CBD occlusion exists, had already been addressed. Therefore, a CT scan was not performed because it would not alter the patient’s care or provide information relevant to the surgical decision. Regardless of CT imaging, cholecystectomy was already indicated given the patient experienced symptomatic gallstone disease with recurring ED visits and evolving biochemical cholestasis.

The pathogenesis of disease remains incompletely understood. Several mechanisms have been proposed in the literature, involving spontaneous thrombosis and ischemia of the HRL [3–5], digestive tract infection via a permeable umbilical vein [1], or inflammation spreading from adjacent organs [6]. In our case, we hypothesize a combined infectious–ischemic mechanism related to recurrent omphalitis and postpartum hypercoagulability. The patient had reported recurring episodes of purulent, malodorous umbilical discharge following her caesarean section, suggestive of untreated omphalitis. The HRL maintains continuity with paraumbilical venous and lymphatic systems, illustrated in Fig. 2, which demonstrates a potential anatomical pathway for translocation of microorganisms. Thereby, ascending infection from postpartum omphalitis represents a plausible mechanism. Concurrently, the postpartum state is characterized by physiological hypercoagulability [9]. Venous stasis and thrombosis of the paraumbilical veins may have led to ischemia, resulting in necrosis and inflammation of the ligament. We hypothesize that the infectious process, combined with thrombosis of the small veins surrounding the ligament, contributed synergistically to its necrosis and associated inflammation. This interpretation is supported by our histopathological findings of suppurative inflammation and fat necrosis and is consistent with mechanisms suggested in previous reports, although infection originating from omphalitis has rarely been emphasized.

**Figure 2:**
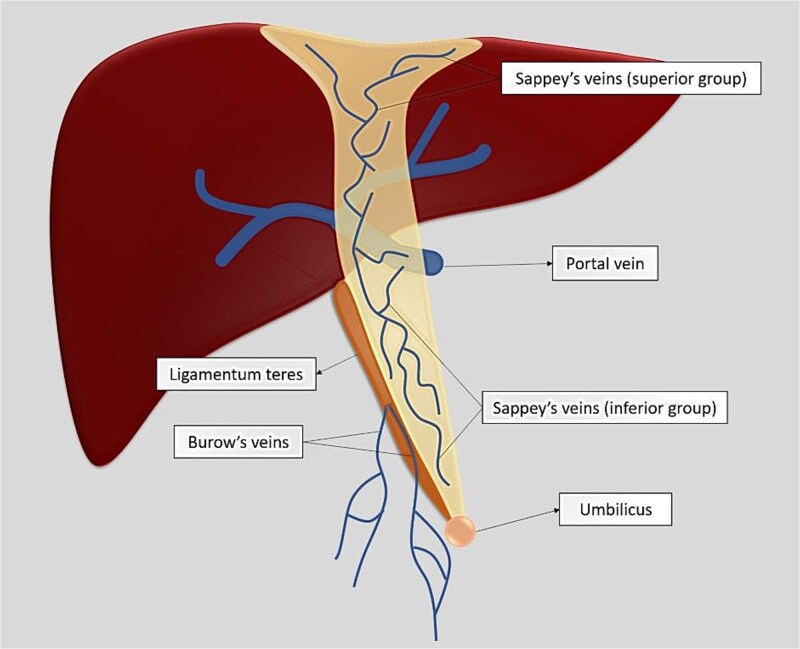
Schematic illustration of the paraumbilical venous network surrounding the ligamentum teres hepatis, demonstrating a potential anatomical pathway for ascending infection [8].

In terms of therapy, surgical excision of the necrotic ligament remains the treatment choice. Laparoscopy is both diagnostic and curative and can be used successfully when anatomy allows; nevertheless, Triki *et al*. emphasize that conversion to open surgery is appropriate when inflammation distorts the hepatobiliary space or obstructs safe dissection, highlighting the importance of operative judgment and adherence to safety principles. In our situation, converting to open surgery allowed for safe dissection when unexpected diffuse inflammation and numerous caesarean-section-related adhesions interfered with identification of essential anatomical structures. The prognosis is excellent, with rapid symptom resolution and no reported recurrences following excision, as supported in the literature review carried out by Bhatt *et al*.

This case adds valuable insight to the limited literature on HRL necrosis in several respects. First, it demonstrates the coexistence of symptomatic gallstone disease and HRL necrosis. Unlike most previously reported cases in which HRL necrosis mimicked biliary pathology in the absence of an established hepatobiliary diagnosis, our patient had objective evidence of concomitant gallstone disease, including documented cholelithiasis, recurrent biliary colic, and histological findings of chronic cholecystitis. Although the biochemical derangements were more readily explained by biliary pathology, the extensive inflammatory and necrotic changes of the ligament suggest that HRL necrosis constituted a clinically significant concurrent process rather than an incidental operative finding. Moreover, while previous reports have suggested ischemic or thrombotic causes, an umbilical source of infection in the postpartum period has yet to be documented, despite the ligament’s anatomical connection to the umbilicus. Our case raises the possibility of an infectious pathway originating from omphalitis, in the setting of postpartum hypercoagulability, providing a plausible combined infectious–ischemic mechanism for HRL necrosis. Finally, our case further highlights the diagnostic challenges associated with HRL necrosis. Our study presents a distinct scenario in which the operative indication was driven by symptomatic gallstone disease with biochemical abnormalities, while the HRL necrosis was an unexpected but clinically significant surgical finding, emphasizing the importance of intraoperative vigilance.

## Conclusion

HRL necrosis is a rare and diagnostically challenging condition. This case demonstrates that HRL necrosis may coexist with symptomatic gallstone disease and be discovered unexpectedly during surgery. While the patient’s biochemical abnormalities were more consistent with biliary pathology, the extensive inflammation and necrosis of the ligament represented a clinically significant concurrent process. Surgical exploration remains the most reliable diagnostic and therapeutic approach.
